# Control of limb loading during active horizontal perturbations at moderate and fast trots in rats

**DOI:** 10.1038/s41598-025-31315-4

**Published:** 2025-12-09

**Authors:** Emanuel Andrada, Martin S. Fischer, Heiko Stark, Dirk Arnold

**Affiliations:** 1 Institute of Zoology and Evolutionary Research, Friedrich-Schiller-University Jena, Jena, Germany; 2 Klinik für Hals-, Nasen- und Ohrenheilkunde, Universitätsklinikum Jena, Jena, Germany

**Keywords:** Rat locomotion, Perturbed locomotion, Control of locomotion, Ground reaction forces, Biomechanics, Mechanical engineering

## Abstract

To understand how small animals cope with complex, unstructured, and unpredictable substrates, we analysed the kinetics of female rats ($n = 10$) moving at a fast and at a moderate trot over an unperturbed substrate and a substrate subjected to active horizontal perturbations. Perturbations were active single forwards or backwards displacements of an instrumented platform by 5 mm or 10 mm amplitudes in 0.05 s. Single leg ground reaction forces (SLGRF) were collected for unperturbed and perturbed locomotion (hindlimbs: 50/102, forelimbs: 45/130, respectively). When negotiating horizontal perturbations, rats displayed gait resetting (braking, accelerating) and non-resetting behaviours. Feedforward strategies differed between the fore- and hindlimbs. In circa 60% of the perturbed trials, forelimbs started the step in acceleration mode, while hindlimbs began the stance mostly in non-resetting mode ($\sim$45%). In about 50% of all perturbed steps, the impulse provided by the SLGRF displayed a change in behaviour according to the expected response to the perturbation. The remaining 50% retained the feedforward strategy. Still, most perturbed trials displayed changes in SLGRF patterns that indicated passive and active reactions to platform shifts. Our results indicate that rats’ sensorimotor control system tunes fore- and hindlimbs differently in expectation of a perturbation. In addition, the tendon-muscle systems of the limbs are recruited to prevent leg collapse at the beginning and end of the stance. At lower speeds, spinal and/or higher centre commands have enough time to re-adapt limb behaviour. At higher locomotion speeds, rats rely more on their limbs’ intrinsic stability and feedforward control.

## Introduction

Small animals can cope with complex, unstructured, and unpredictable substrates easily by adaptively combining the intrinsic stability of their body with neuronal control^1–3^. Small animals must negotiate natural, inherently rough terrains at high speeds in the wild. This leads to very short stance times to re-adapt limb load and thus posture after perturbations. Accordingly, it has been shown that animals negotiating uneven terrains preadjust limb kinematics and impedance before touch-down to reduce the need for neuronal feedback control^3–9^. Similarly, after a sudden drop, running birds’ muscular force response is explained by intrinsic tendon-muscle properties rather than by neuronal modulation^7–10^. Intrinsic stability plays an important role in balance during running when a limb’s touchdown (*TD*) occurs immediately after a perturbation. However, whether and how animals adapt leg load to negotiate perturbations arising during the stance phase has yet to be explored. Characterising how weight-bearing limbs respond to external perturbations during locomotion may help to confirm the importance of intrinsic stability and/or to identify other neuromechanical control strategies for agile and robust locomotion.

In the present paper, we analyse the kinetics of rats moving at moderate and fast trots over unperturbed and actively horizontally perturbed substrates. To obtain the necessary data, we used a novel platform for neuromechanical experiments called ‘the shaker’, which can collect ground reaction forces during active perturbations^11^. Measuring ground reaction forces (*GRF*) is a sensitive and non-invasive way to analyse the contribution of limbs to weight bearing, propulsion and stability^12–16^. Previous work has shed light on level^17^, inclined^18^, and uneven locomotion in rats. Other studies have helped better to understand differences in leg coordination between healthy rats and (a) rats after cortical tract lesions^13^, (b) rats after spinal cord injury^19^, and (c) hemi-Parkinsonian rats^12^. No studies have analysed horizontal substrate shifts during rat or any other small mammal locomotion.

**Fig. 1:**
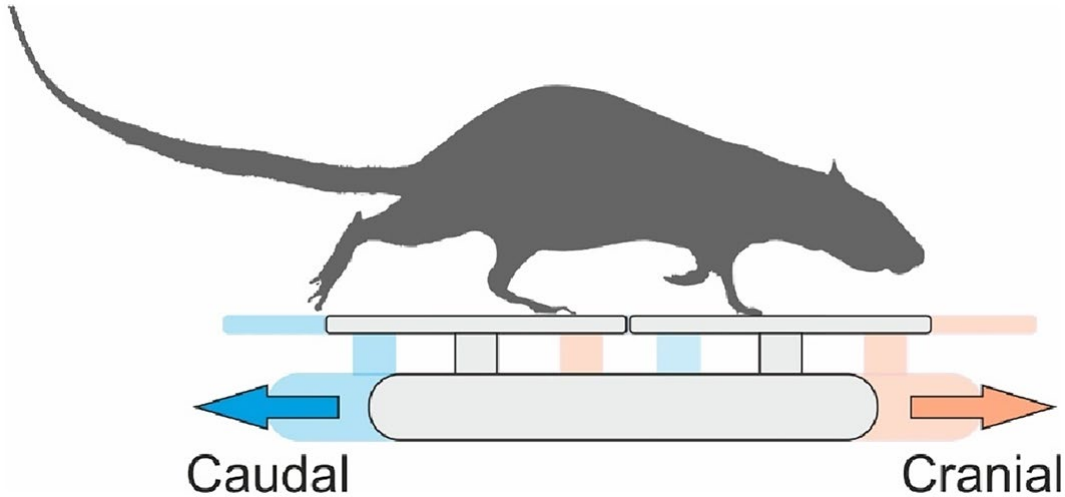
Active horizontal perturbations. The rats experienced random fast horizontal substrate shifts in two directions. During caudal perturbation, the platform moved backwards 5 mm (Cau5) or 10 mm (Cau10) in 50 ms. During cranial perturbation, the platform moved forwards 5 mm (Cra5) or 10 mm (Cra10) in 50 ms.

We collected *GRF*s from rats moving at moderate and fast trots over an unperturbed and an actively horizontally perturbed platform. Backwards motions of the platform were termed caudal perturbation, and forward motions of the platform cranial perturbation (see Fig. 1 and methods for further information). Since we were interested in the response to active horizontal perturbations, we compared vertical and fore-aft forces exerted during unperturbed locomotion to forces exerted in response to perturbations.

We expected that both the amplitude of the horizontal perturbation and locomotion speed would influence sensory feedback and vestibular control. We know we cannot separate the effects of these afferent and descending signals on leg control. However, animals and humans suppress vestibular input during fast running and rely instead on spinal control modulated by type Ia sensory fibres^20–22^. In addition, group I afferents and hip angle extension trigger the transition between stance and swing phase^23–27^. Thus, for slower locomotion speeds and larger perturbations, we expected limb load and contact times (*CT*) to differ (significantly in the case of *CT*) from those observed in unperturbed locomotion. We expected less significant deviation from unperturbed locomotion, independent of perturbation type, for the fastest trials. This last assumption is also based on the inherent stability of the highly automated quadrupedal fast trot.

## Results

We collected locomotion data from ten rats during unperturbed locomotion and from nine during horizontal perturbations (two directions: cranial and caudal, and two amplitudes: 5 mm in 0.05 s and 10 mm in 0.05 s, see Fig. 1). We collected 50 steps for the hindlimbs during unperturbed and 102 during perturbed locomotion. For the forelimbs, we obtained 45 steps during unperturbed and 130 steps during perturbed trials (see Table 1). Rats displayed gait resetting (braking, accelerating) and non-resetting behaviours when coping with horizontal perturbations. In non-resetting trials, the shape and amplitude of the fore-aft limb forces ($GRF_{fa}$) were like the mean curve of the $GRF_{fa}$ during unperturbed locomotion. A few rats stopped the first time they experienced a perturbation (which was not included in our results). After one or two perturbed trials, the rats knew that a perturbation might occur when they stepped on the shaker. Thus, in the following trials, the rats accelerated to cross the perturbing platform. Interestingly, after several trials, many suppressed the accelerating behaviour and performed a non-resetting trot program independent of the perturbation.

### Hindlimbs: perturbed vs. unperturbed locomotion

During unperturbed trot locomotion, the hindlimbs’ contact time (*CT*) was on average 0.177 ± 0.047 s and 0.084 ± 0.013 s for the moderate and fast trot groups, respectively (Table S2). Significant differences were found between the trot groups (moderate vs. fast trot, for all perturbation types, p < 0.05). No significant differences were found between perturbed and unperturbed locomotion, independent of the timepoint of the perturbation onset.

During unperturbed locomotion, the maximum value and the timepoint of occurrence of the vertical component of GRF ($GRF_{v}$) were on average 0.72 ± 0.08 of body weight ($BW = \frac{force}{mass * gravity}$) at 30 ± 7% of the stance for moderate trot, and 0.95 ± 0.04 BW at 35 ± 5% of the stance for fast trot. The maximum value of $GRF_{v}$ differed significantly (p < 0.01) between trot groups, but the timepoint did not. $GRF_{fa}$ exhibited a mean minimum negative value of -0.09 ± 0.03 BW at 12 ± 3% of the stance for moderate trot, and a mean minimum value of -0.08 ± 0.03 BW at 4 ± 3% of the stance for fast trot. The timepoint of the minimum value differed significantly (p < 0.003) between trot groups, but the amplitude did not. During fast trot the maximum positive $GRF_{fa}$ was larger on average (moderate trot: 0.15 ± 0.05 BW vs. fast trot: 0.21 ± 0.07 BW, p > 0.05) and was reached significantly earlier than during moderate trot (moderate trot: 64 ± 8% vs. fast trot: 52 ± 1%, p = 0.0002).

For all early stance perturbations (ESP), regardless of perturbation amplitude and direction, the maximal peak value of the $GRF_{v}$ was larger on average for the fast trot. However, the difference was only significant for cranial translations of 5 mm in 50 ms (Cra5) (Cra5-fast vs. Cra5-moderate, p = 0.046). The peak $GRF_{v}$ value for fast trot occurred later during stance than in moderate trot (p > 0.05). No significant differences were found between unperturbed and perturbed or between perturbed trials (5 mm vs. 10 mm and cranial vs. caudal) in the same trot speed group (moderate or fast). Neither speed nor perturbation type significantly influenced the min and max values or timepoint of the $GRF_{fa}$. For more information, see Supplementary Tables (S2–S8).

However, fast trotting rats exhibited earlier and lower negative $GRF_{fa}$ peak values and later (except for cranial perturbations) and larger maximal positive $GRF_{fa}$ peak values on average than rats trotting moderately. Fast locomotion induced, on average, an earlier transition from braking to accelerating $GRF_{fa}$. A similar earlier transition was observed between moderate and fast unperturbed locomotion.

For late stance perturbations (LSP) of fast trots, the maximum peak value of the $GRF_{v}$ was larger, on average (though only significantly larger in the case of Cau5-fast vs. Cau5-moderate, p = 0.02) and occurred later than during perturbed moderate trots. Maximum $GRF_{fa}$ values and $GRF_{fa}$ timepoints obtained during perturbed locomotion were not significantly different from those obtained during unperturbed trials. However, maximum $GRF_{fa}$ values obtained during caudal perturbations were lower on average than those obtained during cranial ones. For more information, see Supplementary Tables.

**Fig. 2:**
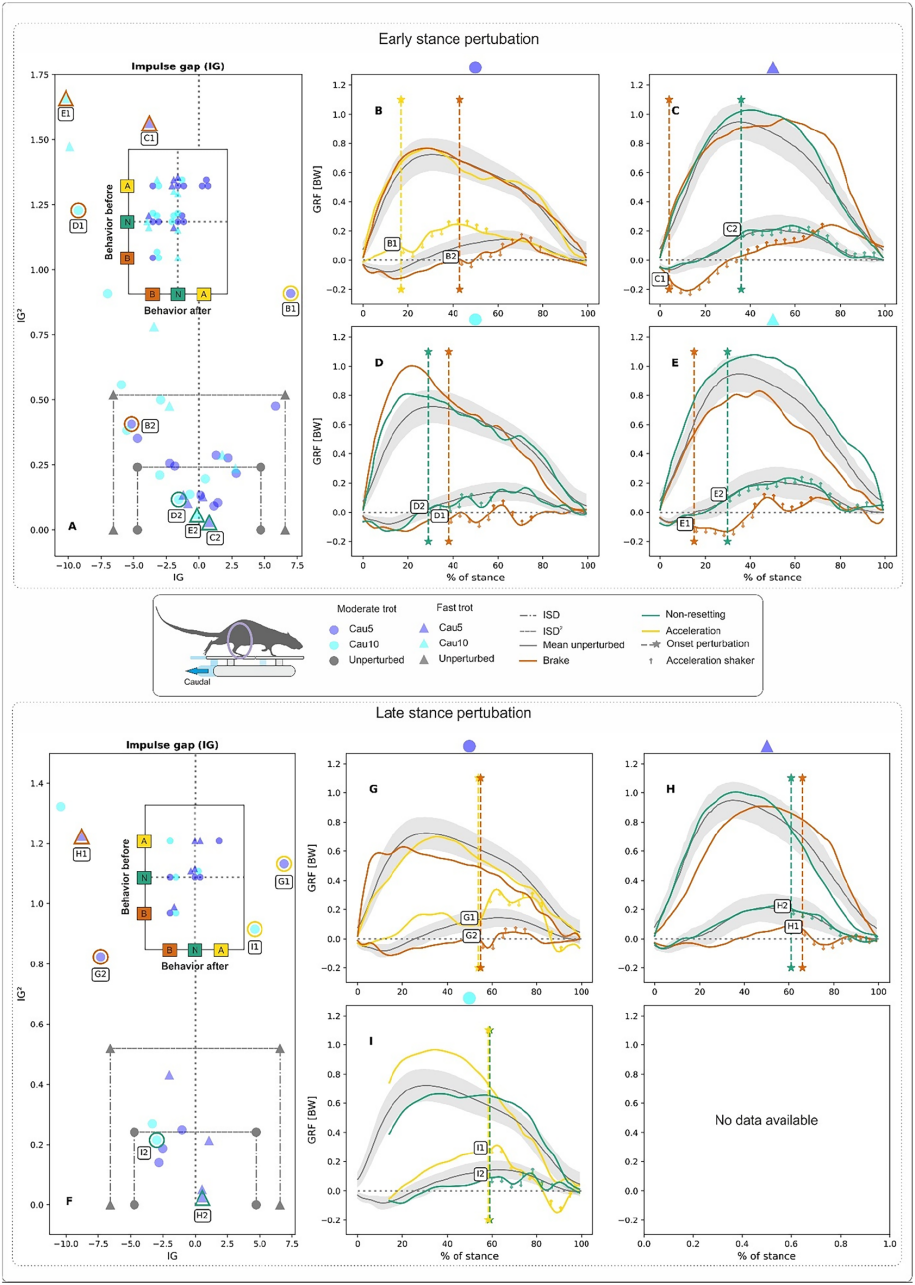
Influence of backwards translations of the shaker (here termed caudal perturbations) on hindlimb kinetics during rat locomotion. Perturbations occurred in the early (**A–B–C–D–E)** and late stance (**F–G–H–I**) phases at moderate and fast trots. Deviations in the patterns of the ground reaction forces (*GRF*) concerning those obtained during unperturbed locomotion were captured in the form of the impulse gap (*IG*) (see **A**,**F**). *IG* measures the shift between perturbed and unperturbed fore-aft GRF during stance. Quadratic *IG* values on the vertical axis of the figures. (**A,F**) Provide a measure of the amplitude of the oscillation of the perturbed fore-aft *GRF* around the unperturbed ones. *IG* and $IG^2$ values relate perturbed locomotion to unperturbed locomotion. Positive values for *IG* indicate that the rats accelerated, and negative ones that the rats decelerated. For non-resetting behaviour, both *IG* and $IG^2$ values must lie in the area formed by the vertical and the horizontal dot-dashed lines with markers (circle for moderate and triangle for fast trot). The vertical lines represent the sum of the standard deviation (SD) of the *GRF*s from unperturbed trials, referred to as the impulse margin (*ISD*). In contrast, the horizontal line represents their quadratic value, $ISD^2$ (**A,F**). The map of behaviours before and after the perturbation is shown in the plots embedded in (**A,F**). The diagonal from bottom left to top right displays pure behaviours. B-C-D-E-G-(**H–I**) display selected examples of *GRF* from perturbed trials (marked in (**A,F**) with circles or triangles, subplot letter and curve number) superimposed on the average profiles ± one SD of *GRF*s from unperturbed trials. Vertical dashed lines with stars in (**B–C–D–E–G–H–I**) denote the onset of the perturbation. Note that the shaker’s horizontal shifts have acceleration and deceleration ramps. These ramps, with a duration of circa 25 ms each, are marked with arrows in the fore-aft *GRF* curves of the perturbed trials in (**B–C–D–E–G–H–I**). Cau5: 5 mm backwards shifts of the platform, and Cau10: 10 mm backwards shifts of the platform, both in 0.05 s).

The combination of the impulse gap (*IG*) and the square of *IG* relates the kinetic behaviour of a perturbed rat limb to the average kinetic behaviour observed during non-perturbed locomotion (see methods). Three related behaviours are possible: acceleration, braking and non-resetting. We analysed *IG* and $IG^2$ combinations for the whole stance, and for the stance periods located before and after the onset of the perturbation. *IG* and $IG^2$ values for the hindlimb’s whole stance phase indicated that rats prefer two strategies when negotiating caudal perturbations: (a) braking and (b) a non-resetting behaviour. Perturbed hindlimbs braked in about 40% of ESP trials and 35% of LSP trials and displayed non-resetting behaviour in roughly 50% of both the ESP and LSP trials (Fig. 2A, F, Table S10). When mapping *IG* and $IG^2$ before and after the perturbation (plot embedded in Fig. 2A, F), we found that 42% of the perturbed steps started in acceleration mode while about 45% started in non-resetting mode. Most of the steps that began in acceleration mode ($\sim$57%) turned non-resetting after the perturbation. The rest remained accelerative (pure acceleration) or braked after the perturbation, in equal proportions (about 21% each). From the steps that started in non-resetting mode, circa 50% remained so, while the other half slowed down. With only one exception, the few steps that began in braking mode remained in that mode (see Fig. 2A).

Figure 2B-C-D-E-G-H-I display selected examples of *GRF*s from perturbed trials superimposed to the average profiles ± SD of *GRF*s from unperturbed trials. In Fig. 2B-C-D-E, responses to ESP can be seen. After the onset of the perturbation, the curves of the $GRF_{fa}$ generally display a dip followed by an increase in the fore-aft forces. At a moderate trot, the dip leads to oscillations that continue for several periods (e.g., Fig. 2D, curves D1 and D2). At a fast trot, on the other hand, $GRF_{fa}$ oscillations are significantly lower, especially in trials in which non-resetting behaviour was observed (e.g., Figs. 2C–E, curves C2 and E2). Responses to LSP display similar patterns to those found in responses to ESP (Fig. 2B-C-D).

**Fig. 3:**
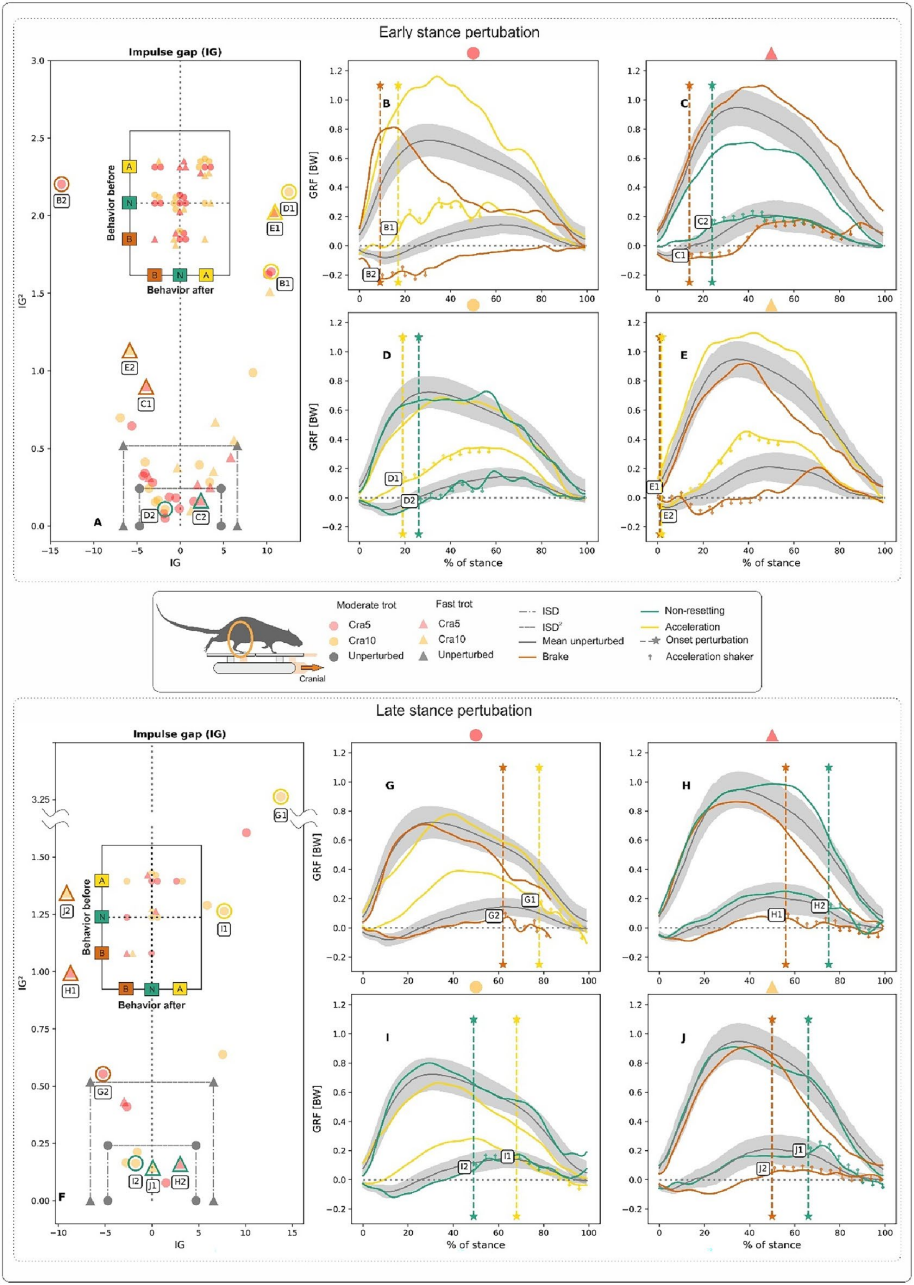
Influence of forward translations of the shaker (here termed cranial perturbations) on hind limb kinetics during rat locomotion. Perturbations occurred in the early (**A-B-C-D-E**) and late stance (**F-G-H-I**) phases at moderate and fast trots. Deviations in the patterns of the ground reaction forces (*GRF*) concerning those obtained during unperturbed locomotion were captured in the form of the impulse gap (*IG*) (see **A,F**). The map of behaviours before and after the perturbation is shown in the plots embedded in **A,F**. (**B-C-D-E-G-H-I**) Display selected examples of *GRF* from perturbed trials superimposed on the average profiles ± one SD of *GRF*s from unperturbed trials. Cra5: 5 mm forward shifts of the platform, and Cra10: 10 mm forward shifts of the platform, both in 0.05 s). See Fig. 2 for further information.

When negotiating substrate shifts in a cranial direction, rat hindlimbs displayed more pure accelerative behaviour than when negotiating substrate shifts in a caudal direction (see quadrant A-A in plot embedded in Fig. 3A). However, in 60% of the trials, rats coped with perturbations without engaging pure accelerating or braking strategies. Pure braking was seldom observed (quadrant B–B in plot embedded in Fig. 3A–D). Similarly, accelerating responses after the onset of the perturbation in trials that started in braking or non-resetting modes were infrequent (B–A quadrant or N–A region in plot embedded in Fig. 3A–D).

Cranial early stance perturbations did not significantly influence the shape of the $GRF_{v}$. However, Fig. 3D displays one trial in which non-resetting behaviour was observed. In this example, a bump can be seen in the $GRF_{v}$ at the beginning of the decelerative part of the perturbation (Fig. 3D, curve D2). In trials in which non-resetting was observed, the $GRF_{fa}$ displayed a bump after a cranial late stance perturbation (see e.g., Figs. 3H and 3J, curves H2, and J1, respectively). At the same time, the $GRF_{v}$ did not show late stance perturbation-related changes. As observed for caudal perturbations, responses in $GRF_{fa}$ after cranial late stance perturbations when rats moved at a fast trot seem more dampened.

### Forelimbs: perturbed vs. unperturbed locomotion

During unperturbed trots, the contact time of the forelimbs was 0.182 ± 0.05 s and 0.09 ± 0.01 s (p < 0.0001) on average for moderate and fast trot groups, respectively. Significant differences (p < 0.05) were also observed in perturbed trials between the contact times of the two gait groups (moderate vs. fast trot. However, within trot groups (moderate or fast), our results showed no significant differences in contact times between perturbed and unperturbed locomotion, regardless of the timepoint of the perturbation onset. For more information, see Table S2 and other Supplementary Tables.

During unperturbed locomotion, the maximal value of the vertical component of GRF ($GRF_v$) and its point of occurrence were on average 0.7 ± 0.1 BW at 59 ± 14% of the stance and 0.94 ± 0.1 BW at 54 ± 7% of the stance for moderate and fast trot groups, respectively. The maximal value of the *GRF* differed significantly between trot groups (p < 0.001). The fore-aft component of the GRF ($GRF_{fa}$) exhibited a mean minimal negative value of -0.096 ± 0.03 BW at 24 ± 7% of the stance for moderate trot and a mean minimal value of -0.081 ± 0.02 BW at 25 ± 14% of the stance for fast trot. Differences between trot groups were not significant. The maximal positive value of $GRF_{fa}$ was roughly the same for both trot groups, (moderate trot: 0.099 ± 0.02 BW vs. fast trot: 0.09 ± 0.02 BW), but during fast trot, the maximal value was reached somewhat earlier (moderate trot: 81 ± 4% vs. fast trot: 74 ± 4%) in the stance phase. Differences between trot groups were not significant.

For early stance perturbations, the fast and moderate trot groups formed two separate data clusters as regards the $GRF_{v}$ (see Table S7). Regardless of the perturbation type, the maximal peak $GRF_{v}$ values were significantly larger (p < 0.05) and occurred earlier during stance (p > 0.05) during the fast trot than during the moderate trot. However, no significant differences were found within trot speed groups (moderate or fast) when comparing unperturbed with perturbed trials or when comparing the different perturbation scenarios. During perturbed locomotion, minimal and maximal peak $GRF_{fa}$ values and timepoints did not differ significantly between trot speed groups (moderate vs. fast). Nor did they vary considerably between the different perturbation scenarios or between perturbed and unperturbed locomotion. For more information, see Supplementary Tables.

For late stance perturbations, maximum $GRF_{v}$ values differed significantly between the two trot groups (p < 0.05). Regarding timing, peak maximal force was reached earlier during the fast trot, but results did not differ significantly between the trot groups (p > 0.05). In perturbated trials, maximum $GRF_{fa}$ values were larger on average when perturbations occurred late in the stance phase. However, neither the maximal values nor the timepoint of their occurrence differed significantly from unperturbed locomotion or between the different perturbation scenarios (p > 0.05). For more information, see Supplementary Tables.

Some differences emerge between the forelimbs and the hindlimbs in their response to caudal and cranial perturbations. When the stance phase is analysed in trials with caudal shifts, *IG* and $IG^2$ reveal that forelimbs accelerated in 46% and braked in 31% of the runs. Only about 23% of the perturbed steps were purely non-resetting.

**Fig. 4:**
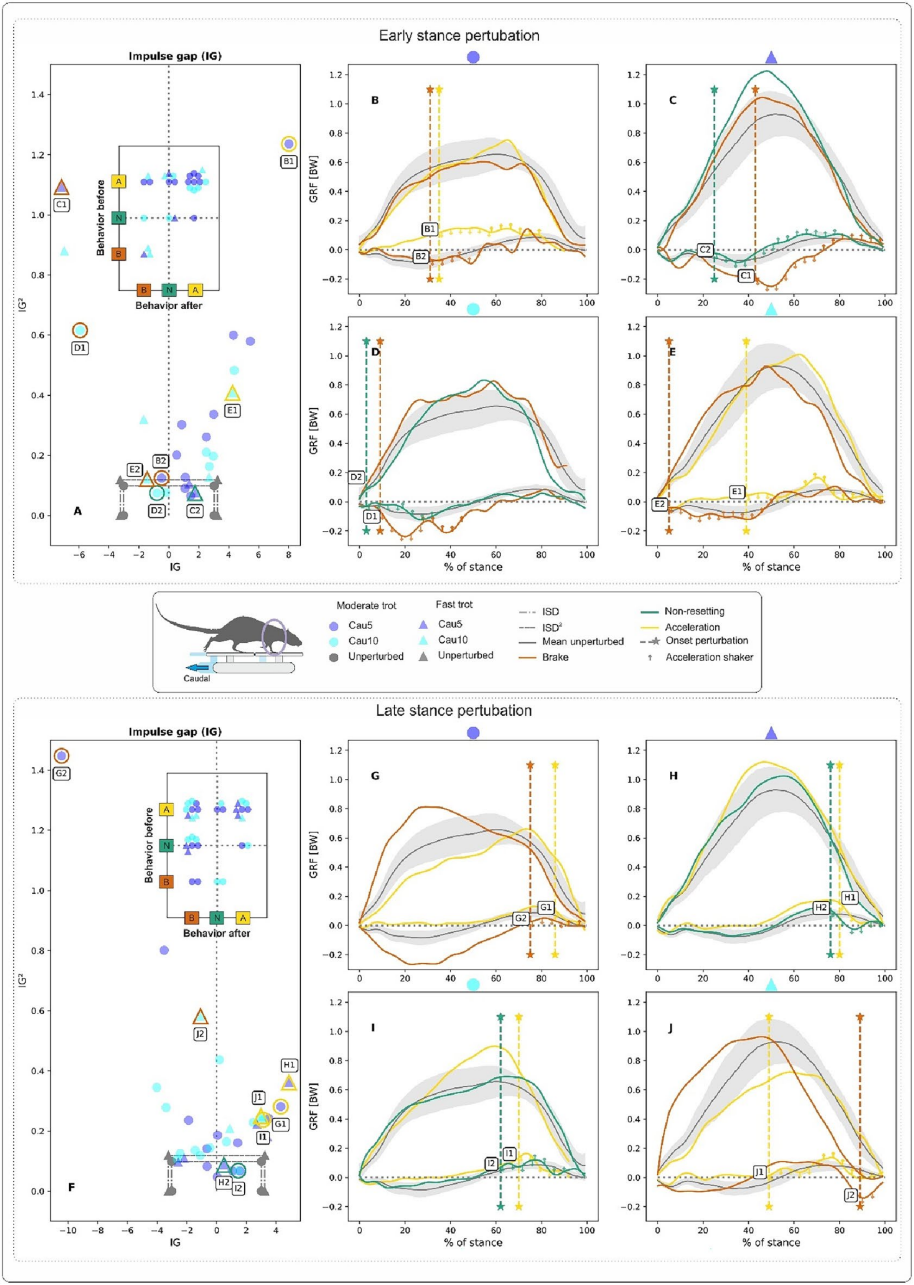
Influence of backwards translations of the shaker (here termed caudal perturbations) on forelimb kinetics during rat locomotion. Perturbations occurred in the early (**A-B-C-D-E**) and late stance (**F-G-H-I**) phases when rats moved at moderate and fast trots. Deviations in the patterns of the ground reaction forces (*GRF*) concerning those obtained during unperturbed locomotion were captured in the form of the impulse gap (*IG*) (see **A,F**). The map of behaviours before and after the perturbation is shown in the plots embedded in (**A,F**). (**B-C-D-E-G-H-I**) Display selected examples of GRF from perturbed trials superimposed on the average profiles ± one SD of *GRF*s from unperturbed trials. Cau5: 5 mm backwards shifts of the platform, and Cau10: 10 mm backwards shifts of the platform, both in 0.05 s). See Fig. 2 for further information.

Before a caudal shift, most of the steps started in acceleration mode ($\sim$62% of all ESP and LSP trials). Circa 57% of ESP and 40% of LSP steps remained purely accelerative, although the logical response to a caudal shift would be to brake (see quadrant A–A in the embedded plots in Fig. 4A, F). However, the percentage of trials in which the forelimbs abandoned the acceleration mode after the onset of the perturbation increased significantly from ESP to LSP (from 15% to almost 50%, respectively).

Purely non-resetting steps were sparse. In addition, 60% of the steps that started as non-resetting shifted into braking mode after the perturbation. This was more marked for the LSP trials (see region N-B in the embedded plot in Fig. 4F and a single trial in Fig. 4H, curve H2). As already mentioned for the hindlimbs, most of the (few) braking steps measured did not change mode after the perturbation (see e.g., region B–B in the embedded plot in Fig. 4A).

**Fig. 5:**
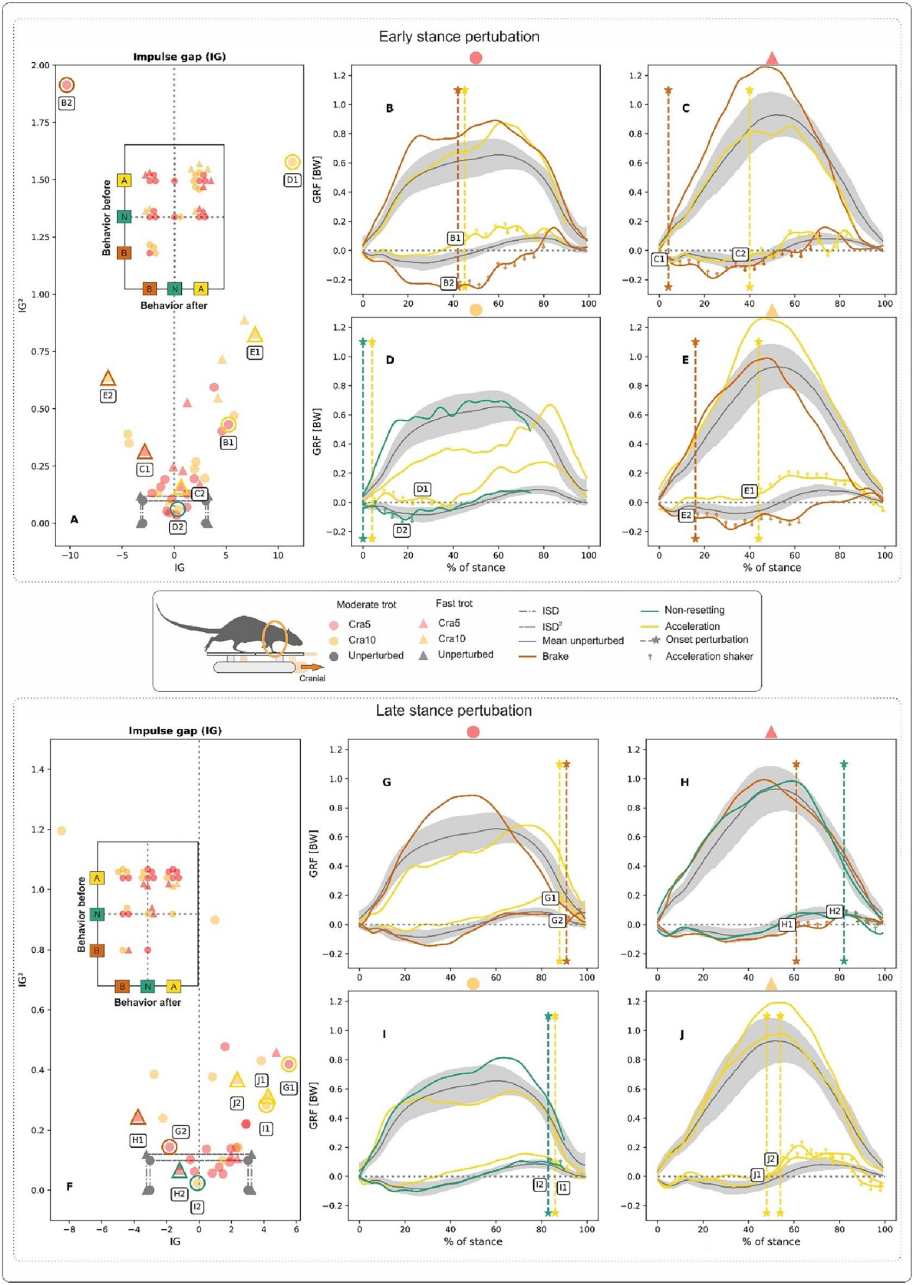
Influence of forward translations of the shaker (here termed cranial perturbations) on forelimb kinetics during rat locomotion. Perturbations occurred in the early (**A-B-C-D-E**) and late stance (**F-G-H-I**) phases at moderate and fast trots. Deviations in the patterns of the ground reaction forces (*GRF*) concerning those obtained during unperturbed locomotion were captured in the form of the impulse gap (*IG*) (see **A,F**). The map of behaviours before and after the perturbation is shown in the plots embedded in (**A,F**). (**B-C-D-E-G-H-I**) Display selected examples of *GRF* from perturbed trials superimposed on the average profiles ± one SD of *GRF*s from unperturbed trials. Cra5: 5 mm forward shifts of the platform, and Cra10: 10 mm forward shifts of the platform, both in 0.05 s. See Fig. 2 for further information.

Forelimb kinetic responses to cranial shifts are presented in Fig. 5. *IG* and $IG^2$ values for the stance phase as a whole indicated that rats’ forelimbs accelerated in $\sim$50% of perturbed steps, while they braked or showed non-resetting behaviour in circa $\sim$25% of the perturbed steps.

The mapping of behaviours observed before and after a perturbation resulted in roughly similar patterns for both ESP and LSP (cp., embedded plots in Fig. 5A, F). In $\sim$60% of the collected steps, rats started by accelerating with their forelimbs. After an ESP, the accelerating forelimb remained in acceleration mode in almost 70% of cases. A shift to braking/non-resetting modes happened only in 25% / 5% of the trials, respectively. After an LSP, forelimbs remained in acceleration mode in 41% of cases and switched to non-resetting or braking modes in 36% and 23% of the trials, respectively.

Only about 30% of the trials that started as non-resetting remained purely non-resetting. Of the remaining 70%, $\sim$40% shifted to braking mode, while the other $\sim$30% shifted to acceleration mode after the onset of the perturbation.

Regarding $GRF_{v}$ and $GRF_{fa}$ patterns, forelimb responses after a caudal/cranial perturbation did not differ from those identified for the hindlimbs. In general, $GRF_{v}$ did not exhibit clear changes after caudal or cranial perturbations. However, rats that were perturbed at a moderate trot and were in acceleration mode displayed a bump in $GRF_v$ at the end of the stand (see Figs. 4B, D, 5B–E). Having said this, the same pattern is sometimes also to be observed in purely non-resetting trials (see Fig. 5I).

A caudal shift often induced a deceleration dip in the $GRF_{fa}$, while a cranial shift induced an acceleration bump/ramp. These dips or bumps/ramps sometimes appeared immediately after the perturbation (e.g., Fig. 4C, D, curves C1 & D1, Fig. 5D, G, curves D1 & G2) and in other cases with a delay (e.g., Fig. 4B, D, curves B2 & D2, Fig. 5B–H, curves B2 & H1). Sometimes, dips or bumps led to oscillations, particularly during a moderate trot (see Fig. 4B, D, curves B2 & D1, Fig. 5B, C, curves B1 and C2). Finally, Fig. 4B (curve B1) shows a trial which displays no clear response to a caudal perturbation.

## Discussion

It is helpful to identify how the system responds to external perturbations to infer the control strategies implemented by a dynamic system. We aimed to establish how weight-bearing limbs respond to horizontal shift perturbations during locomotion. We hoped to shed light on the trade-off between the feedforward and feedback strategies employed during stance that ensure agile and robust locomotion. Feedforward strategies are generated from higher brain centres before the limb contacts the ground and are based on sensory perceptions and internal models. In contrast, feedback modulation of motor output occurs during limb contact and is said to be coordinated via short-latency reflex feedback to spinal circuits^8,28–30^.

To analyse this trade-off, we compared rat single leg *GRF*s during unperturbed locomotion with rat single leg *GRF*s collected during trials where rats had to negotiate rapid horizontal substrate shifts. Based on previous findings^23–27^, we hypothesised that, at lower trot speeds in particular, differences in limb load mediated by Ia afferents might induce differences in contact times compared to unperturbed locomotion. However, we found no significant differences between perturbed and unperturbed locomotion in either speed group. Similarly, statistical methods based on analysis of variance found minimal or no differences in the several control points we proposed to analyse perturbation-related changes to the shape of the *GRF*s (see “Methods”). At the same time, upon visual inspection of the shape of the *GRF* from perturbed trials, clear differences to the mean curves of unperturbed runs are apparent.

Using the impulse gap (*IG*, see methods), we were able to categorise limb responses to perturbations, regardless of amplitude, direction and timepoint of the perturbation, and regardless of trot speed, into three main behaviours: (1) braking, (2) acceleration, and (3) non-resetting.

Rats did not always display the same behaviour to negotiate the same horizontal perturbation. The first time they experienced a perturbation, they usually stopped (not included in our statistical results). Once they realised the platform might move, they exhibited resetting behaviours (slowed down or sped up) to cross the perturbing platform. Interestingly, rats suppressed the accelerating behaviour after a while and performed non-resetting trot programs during perturbed trials^31^. Thus, our results include a ’learning’ effect on the trade-off between feedforward and feedback control.

### Feedforward strategies

Feedforward strategies are planned behaviours (acceleration, braking, non-resetting) before an expected perturbation. Higher centres, such as the cerebellum, generate goal-directed movements based on sensory information and internal models before the leg performs a step on the shaker^32–34^.

Feedforward strategies differed between fore- and hindlimbs when rats faced an expected perturbation. In circa 60% of the trials, forelimbs started the step in acceleration mode, while hindlimbs began the stance phase mostly in non-resetting mode ($\sim$45%). Our results for unperturbed locomotion showed that the standard deviation (SD) for the forelimbs was significantly narrower than that computed for the hindlimbs (for both speed categories, p < 0.0001, cp. e.g., Fig. 2B with Fig. 4B). This phenomenon makes it more difficult for the *IG* and $IG^2$ values to fit inside the non-resetting region for the forelimbs than for the hindlimbs. However, a narrow SD may indicate tighter and more precise loading control in the forelimbs, which might be powered by their ability to manipulate objects. If so, our results suggest that the forelimbs might be more likely to be tuned in expectation of a perturbation than the hindlimbs.

### Pure behaviors

We termed ’pure’ behaviours those that did not change in the face of a perturbation, regardless of its type or the timepoint of its onset. Pure behaviours seem to be governed by strong feedforward strategies.

Pure braking steps occurred seldom. For both fore- and hindlimbs, they were observed in less than 9% of all perturbed runs. This may be connected to the fact that, as outlined above, braking was uncommon as a feedforward strategy. Pure accelerating steps were the most usual control strategy for the forelimbs, but were little observed for the hindlimbs (about 41% and 15% of all perturbed trials, respectively). Note that on average, rat forelimbs braked until about 55%-60% of the stance phase during unperturbed locomotion (see Fig. 4 or 5). Thus, an accelerating impulse at the beginning of the stance configures a completely different motor control set-up. Retractor muscles in the most proximal joints must be recruited from the start of the stance phase.

The occurrence of pure accelerating steps decreased for LSP in both hind- and forelimbs. The most obvious explanation for this would seem to be the need to avoid excessive limb retraction to prevent limb collapse, especially when the platform is moving backwards.

As with the feedforward strategies, pure non-resetting steps were more often observed in the hindlimbs than the forelimbs. At the same time, one in every four perturbed hindlimb steps was pure non-resetting, only 5% of perturbed forelimb steps fitted into this category. Moreover, the frequency of occurrence of pure non-resetting behaviour in the hindlimb remained similar regardless of perturbation type or timepoint of onset.

### Behavioural changes induced by perturbations

In more than 50% of perturbed trials, both fore- and hindlimb behaviours changed after the onset of the perturbation. Hindlimb behaviours changed in about 51% / 56% of the trials after caudal/cranial perturbations, respectively, while forelimb behaviours changed in 55% / 51% of the trials. The changes observed corresponded in the main to those expected to counteract the motion of the shaker (caudal perturbation: braking, cranial perturbation: acceleration). After perturbation, hindlimbs converged more frequently towards non-resetting mode, while forelimbs tended more towards braking or accelerating behaviours. Interestingly, while acceleration mode often shifted into braking mode, shifts from braking to acceleration were extremely rare. This finding indicates that braking feedforward strategies are very attractive, so the limb usually remains in braking mode for most of the stance. Having said this, neither this finding nor the fact that in circa 50% of trials limb behaviour did not change after a perturbation means that the rat limb did not react to the horizontal shifts (see next section).

### Short and long latency reactions in fore-aft forces

Even when limb behaviour did not change after a perturbation, the $GRF_{fa}$ often evidenced reactions that deviated from the patterns depicted during unperturbed locomotion. After cranial perturbations, $GRF_{fa}$ exhibited acceleration bumps (e.g., Fig. 3J, curve J1) or ascending ramps (e.g., Fig. 3C, E, curves C1 & E1), while dips (e.g., Fig. 4H, curve H2) or descending ramps (e.g., Fig. 2I, curve I1) were observed after caudal perturbations. We carefully checked every perturbed step analysed because it was impossible to prevent dips and bumps from being introduced via compensation of the forces created during platform motions (see “Methods”). We can confirm that the amplitude of the bumps and dips we discuss here is two or three dimensions larger than the error we observed due to compensation and synchronisation (see Fig. 7).

The acceleration bumps resisted the cranial displacement of the shaker and prevented an uncontrolled protraction/flexion of the limb. The rapid responses observed indicate that cranial perturbations likely stretched the extensor limb muscles (e.g., *M. triceps surae* in the forelimbs and *M. gluteus* in the hindlimbs), triggering, via muscle spindles and fast myelinated Ia afferents, monosynaptic reflexes^35,36^. In some cases, bumps occurred right after the perturbation (see e.g., Fig. 5B, curve B1, Fig. 3J, curve J1). At first glance, this pattern seems to correlate with an expected reaction to the perturbation, but the reaction time was arguably too fast, even for reflex responses. At a conduction velocity of about 45 $\frac{m}{s}$^37^, the time taken for the stimuli to reach spinal centres and return to the limbs would be under 2 ms. However, muscle contraction times in the rat are longer than 10 ms^38^. Thus, sensorimotor control is not likely to explain the force changes. However, if muscles worked isometrically, which is a necessary condition for the pantograph mechanism in quadruped limbs^39^, passive structures such as tendons might help to explain this rapid increase in limb loading. Passive force compensation has, for example, been observed in guinea fowl when negotiating sudden drops^7,40^.

Caudal perturbations induced in most cases a dip in the $GRF_{fa}$ of both fore- and hindlimbs that opposed the retraction of the limb. However, when perturbations occurred early in the stance phase, this reaction occurred later in the forelimbs than after cranial perturbations (e.g., Fig. 4B, curve B2). In other words, the forelimbs retracted for several milliseconds due to the action of the active platform without this reaction being counteracted. One explanation for this finding is the notion that muscle force depends on its state (strain, strain rate, strain history and loading) (see, for example^10,41^). On the other hand, the finding could also indicate a difference in sensitivity tuning between muscle spindles in the primarily silent flexor muscles and the active extensor muscles. Note that the $\gamma$-motoneurons allow spindle response properties to be tuned^35^. There is experimental evidence to show that $\gamma$-efferent commands covary with joint angles during movements^36,42,43^, perhaps to ensure optimal work between muscle extensor/flexor groups^44^. Interestingly, just before *TO*, the dips appear in the forelimbs immediately after a caudal perturbation (Fig. 4H, curves H1 & H2). In this part of the stance, the flexor *M. biceps brachii* is activated to prepare for the swing phase, and the tendons may rapidly work against the hyperextension of the elbow.

Ascending or descending ramps with significant force increments appeared circa 10 ms to 25 ms after perturbation onset (see e.g., Fig. 2E, curve E1, Fig. 3C, curve C1, Fig. 5B, B1). Note that in our experiments, the rat moved with the platform; the perturbation was not limited exclusively to the limbs. Thus, due to the short stimulus distances in rats, spinal control and/or higher centres like the vestibular system might have been involved in the observed responses. Motor commands from higher centers are not only transmitted to the leg’s central pattern generators (CPG) via descending spinal pathways^8,26,28,30,45,46^, the lateral vestibulospinal tract also has direct connections to flexor and extensor motoneurons at the knee and the ankle joints. Thus, it can modulate muscle activity and, in some cases, can affect the timing of the rhythm^47^.

### Influence of trot speed

In our experiments, the rats trotted at self-selected speeds. To reduce the effect of speed on the results, we separated the trials into moderate and fast trots (see methods). As stated above, we found significant differences in *CT* and the shape of the *GRF* between moderate and fast locomotion within each group (e.g., non-perturbed-moderate vs. non-perturbed-fast). However, differences were insignificant when unperturbed locomotion was compared with perturbed locomotion at the same speed category (e.g., non-perturbed vs. Cra10-moderate).

At moderate (slower) trot speeds, the response to the horizontal shift of the shaker frequently led to oscillations in the $GRF_{fa}$. Two possibilities can be considered here. A) The muscles were recruited discretely to negotiate the perturbation, or b) muscle activation became unstable. It is well known that feedback control systems involving muscles as sensor and actuator units can lead to oscillating responses if negative feedback (e.g., inhibitory signals from Renshaw cells, tendon organs, contralateral limbs) experiences a delay^36,48^.

At a fast trot, we found that oscillations in the responses to a perturbation were significantly smaller (see Figs. 2C, E, 4C, curves C2, E2, and C2 respectively). Similarly, ’dampened’ responses were observed during pure accelerative steps (see, e.g., Figs. 3D, curve D1, 4B, curve B1). Higher centres increase $\alpha$ and $\gamma$ activity to enable organisms to move faster. Higher $\gamma$ activation levels, in turn, reduce the sensitivity of the spindles to muscle stretch^35^. In terms of limb size and limb speed, rat joints lie in a viscous overdamped region at a fast trot^49^. In this region, viscous effects dissipate most energy in the joints, and perturbations do not cause the limb to oscillate. As locomotion speed increases, feedback control gain decreases, and rats rely more on the intrinsic stability of their body plan and feedforward control.

### Conclusion

Since quadrupedal mammals maintain ground contact with two legs during the trot, they can balance out horizontal shifts more easily than bipeds. In line with this, our experiments revealed that no single response to a horizontal change exists. In rats, reactions to perturbation seem to result from a complex combination of expectation and experience, as well as the intrinsic stability of the body. With no experience, rats usually stopped after a horizontal perturbation. With experience, rats learned to include feedforward and feedback control, speeding up to cross the perturbing platform. With even more experience, rats often suppressed the accelerating behaviour and performed a non-resetting trot program during perturbed trials^31^. Interestingly, reactions to perturbations differed between the fore- and hindlimbs. The loading of the forelimbs seems to be more strictly controlled than in the hindlimbs. During stance, intrinsic stability and feedback control tune limb-loading according to perturbation type and timepoint of onset, feedforward strategy and experience. When the perturbation occurred at the beginning or end of the stance, the passive properties of the tendon-muscle system of the limb prevented leg collapse. In between, spinal and/or non-spinal centres had enough time to readjust limb behaviour at moderate speeds. At higher speeds, our results indicate that rats rely more on the inherent stability of their limbs and feedforward control.

## Methods

### The shaker

**Fig. 6:**
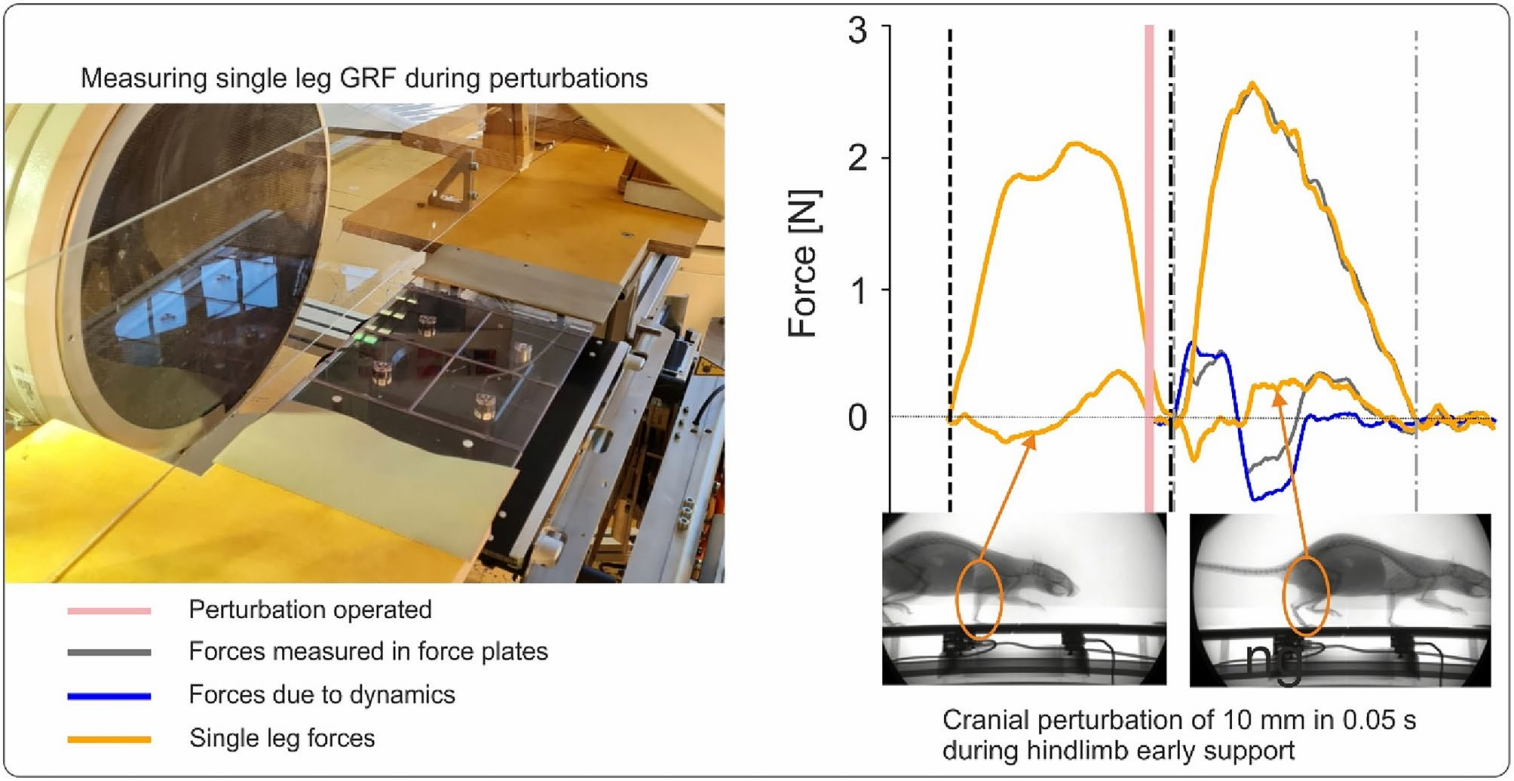
Analysing actively perturbed locomotion in rats. Left) Experimental setup combining biplanar X-ray and an active platform ”shaker” capable of force measurement. Right) ground reaction forces before (right forelimb) and during a horizontal perturbation (right hindlimb). The platform was shifted 10 mm in 0.05 s in a cranial direction at the beginning of the hindlimb support phase. Grey lines represent the forces measured by the plates, blue lines represent the forces produced by the motion dynamics (previously saved), and orange lines represent the forces exerted by the rat.

**Fig. 7:**
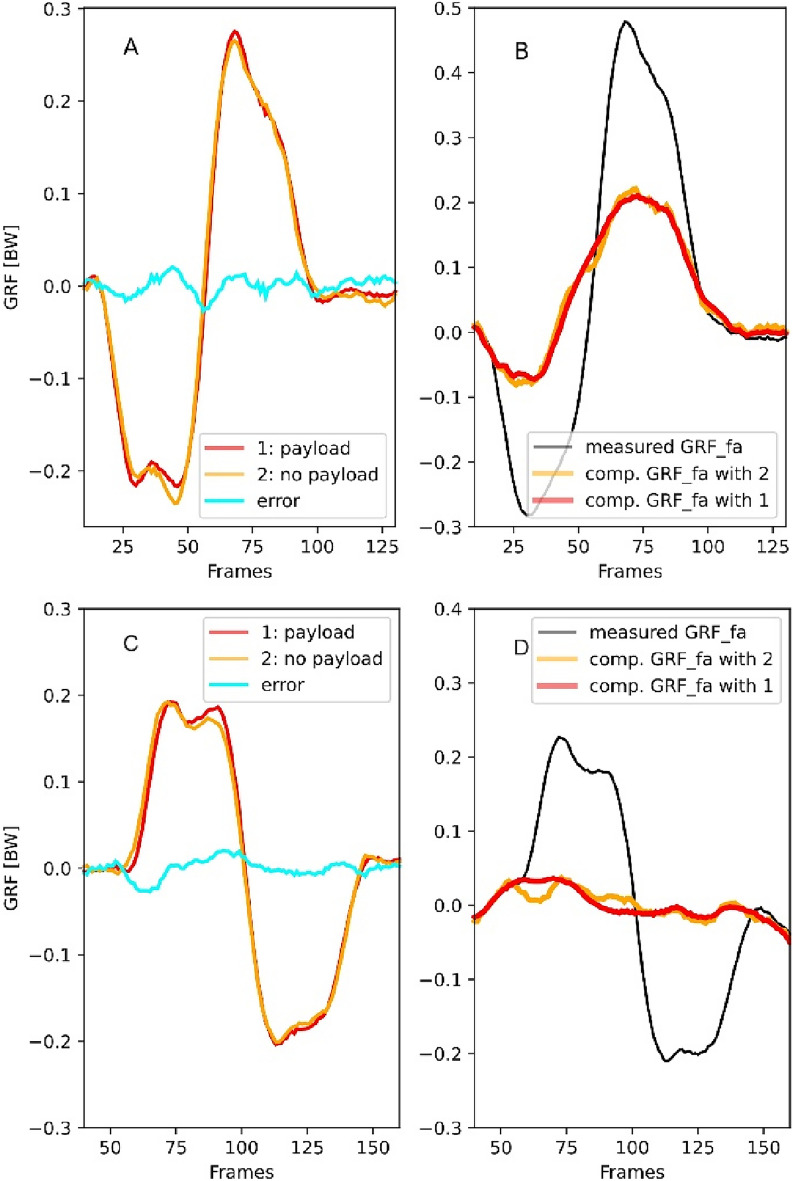
Error in the estimation of the $GRF_{fa}$ obtained by subtracting the force measured by the force plates without payload (orange) from those obtained with the rat on the platform (red). (**A,B**) Caudal perturbation of 10 mm in 0.05 s, Cau10, C-D: Cranial perturbation of 10 mm in 0.05 s. In these trials, the rats (’Red_1’ in (**A,B**), and ’Right’in (**C,D**)) missed one force plate during the perturbation. Thus, that force plate measured only the forces generated by the motion of the shaker (see (**A,C**), red curves). For these trials, it is possible to compute the error ((**A,C**), cyan curves) introduced when using the forces obtained from the displacement of the shaker without payload ((**A,C**), orange curves) to estimate the $GRF_{fa}$. The right panel displays the effect of using both compensation forces on the measured $GRF_{fa}$. Error can produce small bumps/dips or oscillations of maximum 0.03 BW.

The active platform, ”the shaker,” was already introduced in Andrada et al.^11^. The shaker can generate single or combined horizontal, vertical, and tilting perturbations with a payload up to 1 kg. To make it suitable for small animals during striding locomotion, the shaker was conceived to generate horizontal and vertical perturbations with amplitudes of up to 1 cm at oscillation frequencies of up to 10 Hz (repeat accuracy < 0.1 mm). Tilting perturbations are only used in posture experiments and therefore do not need to satisfy such high oscillation rates. The support platform consists of two carbon plates with an elastomer in between (thickness = 6 mm) and two or three carbon tubes (external diameter $\phi$ = 25 mm, wall thickness = 1 mm). On the support platform, a ring made of Polyoxymethylene (POM) was rigged to permit mounting up to four force plates based on ATI-Nano 17®force/torque sensors^50^. Four plates made of acrylic glass (10 cm x 10 cm x 0.5 cm) were used as a platform for collecting *GRF*s and centre of pressure (*CoP*) (see Fig. 6 left). Due to restrictions in the data acquisition software and a reduced x-ray window (see below), we only instrumentalised the left part of the shaker. The two plates on the right side of the shaker have ’dummy’ sensors made of aluminium.

The vertical oscillations of the platform (z-axis) are produced by two linear servomotors (Beckhoff, mod. AL8042). The horizontal oscillations (x-axis) are made by one linear servomotor (Beckhoff, mod. AL8040). The linear motors are controlled by digital compact servo drives (Beckhoff, mod. AX5206 / Beckhoff, mod. AX5106). The maximal translation amplitude ratio is 2 cm in 0.05 s. GUI and control programs were written in Beckhoff’s software environment (Beckhoff TwinCAT). Time-dependent perturbation profiles can be freely designed using comma-separated values (CSV-file). Perturbations can be triggered manually or using a photoelectric sensor. The shaker was constructed by H&S Robotics, Ilmenau, Germany.

### Animals, experiments, and data analyses

**Table 1:** Animals, weights, and collected steps (E: early stance, L: late stance, H: hindlimb, F: forelimb).

Rat name	Weight (g)	Steps unperturbed		Steps Cra5				Steps Cra10				Steps Cau5				Steps Cau10			
Stance				E		L		E		L		E		L		E		L	
Limb		F	H	F	H	F	H	F	H	F	H	F	H	F	H	F	H	F	H
Right	400	11	7	6	4	6	1	1	1	4	2	2	3	3	2	4	2	5	2
Left	290	4	5	3	5	4	2	1	6	6	2	2	7	2	2	1	4	2	1
Black 1	300	4	3	3	0	0	0	2	2	2	0	0	1	2	0	0	1	2	0
Black 2	295	4	3	1	1	1	1	3	2	1	2	1	1	3	2	2	2	0	0
Black 3	303	1	3	1	1	1	0	1	1	1	0	2	1	1	1	1	2	0	0
Black 4	298	4	4	0	0	3	1	1	2	0	0	2	1	1	0	2	1	2	0
Red 1	296	5	5	4	7	3	0	3	2	1	0	2	1	2	1	1	1	1	0
Red 2	301	7	6	4	0	1	0	0	0	0	0	0	0	1	0	1	2	3	1
Red 3	305	3	3	0	0	0	0	0	0	0	0	0	0	1	0	0	0	0	0
Red 4	290	7	6	1	2	4	3	2	3	2	1	3	2	3	2	1	1	1	0

Ten adult female rats (*Rattus norvegicus*) weighing from 250 g to 400 g (see Table 1) moved across a 2.3 m walking track constructed around the shaker. The Committee for Animal Research of the State of Thuringia, Germany, approved the animal care and all experimental procedures (registry number: 02-060/16).

In our experiments, the rats trotted at self-selected speeds. In the first set of experiments, several unperturbed trials were carried out to build a basis for comparison. Like many small prey animals, rats do not necessarily move steadily. For this reason, we favoured the term unperturbed locomotion over steady-state locomotion. In a second set of experiments, the rats randomly experienced: (a) no perturbations, or (b) one-way linear perturbations of 5 mm or 10 mm in 0.05 s when they stepped on the shaker. The experimenter manually triggered the onset of perturbation. Perturbations were vertical (up/ down) or horizontal (forward/backwards). Here we present results from horizontal perturbations. Horizontal perturbations were termed cranial or caudal based on the direction of motion of the platform relative to the animal (see Fig. 1). Cranial perturbation refers to the forward motion of the platform, while caudal perturbation describes the backwards motion of the platform. The duration of perturbation (0.05 s) was chosen to make up about 50% of the stance time during the fast trot in rats. 0.05 s is about five times the conduction delay (time required by a limb to respond to a perturbation) in a rat^37^. Furthermore, the chosen perturbation time is also long enough to take account of other sources of delay in responses to stimuli, such as synaptic transmission and force generation^37,51^. In other words, rats in our experiments had enough time during/after perturbations for neural feedback^10^, including compensation through internal models^52^.

Body and limb kinematics were collected using a biplanar high-speed X-ray fluoroscope (Neurostar, Siemens, Erlangen, Germany) and two synchronised standard live high-speed cameras (SpeedCam Visario g2, Weinberger, Erlangen, Germany). One plane of the X-ray machine recorded the motions of the rat in the sagittal plane (see Fig. 6 left). The second plane, which records typically from above the animal, was rotated $30^{\circ }$ from the vertical position to minimise interference with the force/torque sensors and to improve recognition of the tantalum beads after motion capture. The X-ray machine parameters were 55 kV and 40 mA, with a sampling frequency of 500 Hz. *GRF*s were collected at 1.0 kHz, and force and X-ray data synchronised electronically (post-trigger). Force data were synchronised with the onset of perturbation using a visual signal integrated into the shaker, which was captured by the live high-speed cameras. This synchronisation was necessary to permit a subtraction of the forces induced by acceleration (measured without payload) from those collected during posture/locomotion experiments (see Fig. 6 right). Note that the motion profiles produced by the linear servomotor (Beckhoff, mod. AL8040) were similar whether the rat was on the platform or off it (Fig. 7A–C). Therefore, subtracting the forces obtained without payload from those obtained during actual trials returns a satisfactory approximation of the leg load during perturbations. However, compensating for forces obtained during motion without payload can introduce small bumps or oscillations (we measured a maximum amplitude of 0.03 BW) in the force data (see Fig. 7B, C). The timepoint at which errors are added to the force data depends on the accuracy of the shaker’s motion and the synchronisation error.

Rough force data was filtered using a 7th-order Butterworth low-pass filter with a cut-off frequency of 100 Hz applied in a zero-phase digital filter. Before analysis, filtered data were interpolated to 100 points between *TD* and *TO*. Double support phases occurring on the same force plate were deleted from the interpolated force data. Results were grouped and then exported to cvs-tables based on perturbation type (Cra5: cranial perturbation of 5 mm in 0.05 s, Cra10: cranial perturbation of 10 mm in 0.05 s, Cau5: caudal perturbation of 5 mm in 0.05 s, Cau10: caudal perturbation of 10 mm in 0.05 s), *GRF* components (vertical, fore-aft), speed group based on contact time (*CT*) (moderate trot CT > 110 ms, vs. fast trot CT $\le$ 110 ms), and onset of the perturbation (early stance phase: from 0% to 50% and late stance phase: 51% to 99%). While the speed groups might reduce the effects of speed on the results, the timepoint of onset of the perturbation might roughly reflect the effects of leg orientation and perhaps muscle state in the response to perturbations.

### Statistical analysis

The large database required a detailed analysis with regard to various constraints. In a first step, the data sets were divided into sub-data sets with five perturbation groups: unperturbed, Cau5, Cau10, Cra5, Cra10. These were again divided into two speed categories: moderate and fast trot. This resulted in ten groups for the various trails, which were tested against each other (Table 1).

Since the onset of the perturbation and the timepoint of TD did not always coincide, an additional subdivision was made with regard to these events: before TD (perturbation onset occurred before TD), early TD (perturbation occurred during early stance), during TD (perturbation and TD coincided with a maximal gap of 5 frames), late TD (perturnbation occurred during late stance), after TD (first step after de end of a perturbation) and after TD leg two (second step after the end of a perturbation). In addition, a distinction was made between the spatial axes: vertical, lateral and cranocaudal. These 18 subdivisions were statistically considered for the groups of ten.

For each subdivision, we analysed *CT*s, *GRF*s, the position of certain *GRF*s and moments at several points and for ranges. For the points, measurements were taken at 25%, 50% and 75% of a step. For the ranges, measurements were taken within 0%-50%, 25%–75% and 50%–100% of a step. From this, the minimum, mean, median or maximum of the range was calculated. This resulted in 39 statistical analyses for each of the 18 subdivisions, yielding a total of 702 statistical analyses. Each statistical analysis was carried out in the same way for the groups of ten.

Each statistical analysis was analysed separately for the fore- and hindlimbs. First, an analysis of variance (repeated measures Anova) was performed to test whether the perturbation groups differed significantly (R:aov^53^). Based on this, a post-hoc test (TukeyHSD) was performed to compare all possible groups^54^. As homogeneous variance could not be assumed for all parameters, a variance test (Levene) was also carried out. For problematic variances, all possible groups were compared using a further post-hoc test (Games-Howell;^55^). The statistical analysis was carried out using the freely available software R (version: 4.2.3). The libraries (R.matlab, data.table, Rfast, tidyverse, stats, rstatix, car, ggstatsplot, viridis, openxlsx) were used for specific functions. R-scripts were created using the free program ”master” (https://starkrats.de). This analysis is available on Figshare for all trails and for the trails averaged per rat (https://doi.org/10.6084/m9.figshare.c.8120747).

Because statistical analyses mostly failed to detect differences between unperturbed and perturbed locomotion, we introduced a measure that we termed the impulse gap (*IG*) to classify perturbed trials relative to unperturbed ones. *IG* provides the sum of the distances (the area) between the curve representing the mean value of $GRF_{fa}$ obtained during unperturbed trials (mean $GRF_{fa-Unpert}$) and the $GRF_{fa}$ curve of a perturbed trial ($GRF_{fa-Pert}$) for i frames. Thus $IG = \Sigma ^{n}_{i= 0}$ (mean $GRF_{fa-Unperti} - GRF_{fa-Perti}$). In other words, *IG* measures the shift between perturbed and unperturbed fore-aft *GRF* during the analysed period. We then compared *IG* with the impulse margins (*ISD*) obtained during unperturbed locomotion. *ISD* represents the area computed between the mean $GRF_{fa}$ for unperturbed trials ($GRF_{fa-Unpert}$) and the standard deviation (SD) of $GRF_{fa-Unpert}$, $ISD = \Sigma ^{n}_{i = 0}$ (mean $GRF_{fa-Unperti} - SD~GRF_{fa-Unperti}$). If $IG > ISD$, the leg accelerated during the step/ part of the step relative to the averaged unperturbed step. Conversely, if $IG < -ISD$, the leg brakes. Finally, if $-ISD< IG < ISD$, it means that the impulse gap remained within the impulse value measured between mean $GRF_{fa-Unpert} \pm SD~GRF_{fa-Unpert}$. This is a necessary condition to categorise a step as a non-resetting step. However, a $GRF_{fa-Pert}$ that oscillates around the mean $GRF_{fa-Unpert}$ could, even with larger amplitudes, return very low *IG* values. Therefore, $IG^2$ must also be lower than or equal to $ISD^2$ to categorise a step as non-resetting. We analysed *IG*, $IG^2$, *ISD* and $ISD^2$ for the whole step and the portions of the step before and after the perturbation. Measuring *IG* for the whole step provides information about the general behaviour during the contact phase. Measuring *IG* before the perturbation may provide information about feedforward strategies, while after the perturbation, it provides an insight into the behavioural reaction to the perturbation. For every behaviour, we then inspected individual trials to look for perturbation-related alterations to the shape of the *GRF* curve after the triggering of the horizontal shifts. Data analysis was performed using Python (versions 3.9.7 and 3.11.5) and the following Python libraries: Pandas, Scipy, Numpy, and Pickle. Plots were produced using Matplotlib.pyplot.

## Data Availability

The datasets used and/or analysed during the current study are available from the corresponding author on reasonable request.
